# Mitophagy Balance in Various Cell Subsets in Patients with ANCA-Associated Vasculitis and Correlation with the Presence of Anti-Neutrophil Cytoplasmic Antibodies

**DOI:** 10.31138/mjr.31.3.366

**Published:** 2020-07-04

**Authors:** Aggelos Banos, Konstantinos Thomas, Panagiotis Garantziotis, Nikos Malissovas, Anastasia Filia, Antigoni Pieta, Dionysios Nikolopoulos, Savina Moustafa, Alexandros Panagiotopoulos, Dimitrios T. Boumpas, Dimitrios Vassilopoulos

**Affiliations:** 1 Biomedical Research Foundation of the Academy of Athens, Greece; 2 Joint Rheumatology Program, National and Kapodistrian University of Athens, School of Medicine, Clinical Immunology-Rheumatology Unit, 2^nd^ Department of Medicine and Laboratory, Athens, Greece; 3 Joint Rheumatology Program, National and Kapodistrian University of Athens, School of Medicine-Clinical Immunology-Rheumatology Unit, 4th Department of Medicine, Athens, Greece

**Keywords:** vasculitis, autoimmunity, transcriptomics, mitophagy

## Abstract

ANCA-associated vasculitides (AAVs) are characterised by heterogeneous molecular and pathophysiological traits, causing ambiguous differential diagnosis and taxonomy. Response to therapy has proven far from successful, contributing to high mortality. Transcriptome analysis of different vasculitis subtypes adds new leads in elucidating mechanisms of disease and the role of specific cell subsets to them. Recent findings have shown that mitophagy is a procedure whose imbalance could lead to immune dysregulation with certain involvement to autoimmunity. Inflammatory response related mitophagy is yet to be described in AAVs. We here describe a research protocol to investigate mitophagy in monocytes, neutrophils, and T cells in AAV patients, and the relationship of disturbed mitophagy with ANCA seropositivity.

## INTRODUCTION

ANCA (anti-neutrophil cytoplasmic antibodies) associated vasculitides (AAV) include granulomatosis with polyangiitis (GPA), microscopic polyangiitis (MPA), and eosinophilic granulomatosis with polyangiitis (EGPA) subtypes, being the most aggressive as to morbidity and mortality rates compared to other systematic vasculitides. Worldwide incidence of AAVs is approximately 26/10^6^ individuals, with higher impact at ages of 65 to 74 years. At the same time, epidemiological studies in Greece are sparse, while in Crete, the respective incidence is 19.5/10^6^ individuals, with younger patients presenting GPA, and older ones with MPA.^1^ Despite the intensive therapeutic approaches including corticosteroids and immunosuppressant drugs (cyclophosphamide, azathioprine, methotrexate, mycophenolate mofetil) or their combination, patients suffer from subsequent relapses and high mortality, estimated at approximately 25% in 5 years.^2,3^ Recently, rituximab has been approved as therapeutic solution for induction of remission and maintenance of GPA and MPA, resulting to encouraging outcomes. Independent prognostic factors that are associated to high mortality are advanced age, renal involvement and high activity scores (BVAS and BVAS/WG) at diagnosis. Pathogenetic mechanisms of AAVs are yet to be fully elucidated and there are no specific biomarkers discerning disease subtype, response to therapy, or disease course/relapse rate.

RNA-sequencing is a cutting-edge technology that provides tools for transcriptomic characterisation of the disease and molecular clustering of patients based on their transcriptional profile. A first study of transcriptome profiling defined a transcriptional signature of CD8 T-cell exhaustion, associated with prediction of better prognosis in AAV, along with other autoimmune diseases.^4^ In order to utilise this method to explain disease mechanisms and strengthen standard classification criteria, we analysed whole blood transcriptome of 42 AAV patients (with MPA, GPA or EGPA) and 11 healthy individuals. Gene ontology and enrichment analysis on differentially expressed genes between patients and healthy individuals formed a pan-vasculitis disease signature associated with deregulation of pathways related to neutrophil degranulation, immune activation, and immunometabolism among others. Distinctive gene pathways can discriminate different AAV subtypes from healthy state, giving molecular insights on differential diagnosis. Principal component analysis proved that patients with renal involvement in AAV exhibit a discrete transcriptional profile from those with SLE nephritis. Other pathways emerging from intra-disease comparisons are deregulation of cell cycle, DNA damage response, and mitophagy. We sought to study this last procedure and examine its correlation to ANCA seropositivity.

Mitochondria are fundamental effectors of immune signalling as well as rheostats of energy and ROS production. Their malfunction could result to pathology related to cytokine signalling. Mitophagy is a homeostatic mechanism that drives destroyed or redundant mitochondria to autophagy pathway, playing an important role in immune balance.^5,6,7,8,9^ Recent studies in inflammatory bowel disease (IBD) and systemic lupus erythematosus (SLE) prove that mitophagy links autophagy with inflammatory response.^10,11,12^ The importance of the role of mitophagy in vasculitis has yet to be clarified.

## AIM OF THE STUDY

Comparison of mitophagy in different cell subtypes in AAV patients and correlation with ANCA positivity. Utilisation of mitophagy pathway to assess cell phenotypes associated with disease activity and vasculitis subtype.

## METHODS

Based on the initial transcriptomic data, we plan to assess mitophagy through 4 different directions.

In work package 1, validation of transcriptional profile for mitophagy genes will take place in an independent cohort of 10 patients (n=5 with ANCA+ and n=5 with ANCA−). Recruitment criteria will include active disease (BVAS>0) and whole blood will be collected. RNA extraction will be followed by cDNA synthesis and qPCR for a specific panel of mitophagy related genes.

In work package 2, collection of different cell subtypes from the same patients (WP1) and measurement of their frequencies will be performed by either magnetic isolation or flow cytometry. Whole blood will be processed through double density Ficoll gradient, in order to isolate PBMCs and neutrophils. Flow cytometry will be exploited to assess the frequencies and purity of monocytes/macrophages (CD14, CD16), CD8^+^ T cells and neutrophils (CD66b, CD15). The latter neutrophilic population will be collected through FACS (fluorescence-activated cell sorting) while monocytes and CD8 T cells will be isolated through specific magnetic columns in high purity.

In work package 3, levels of expression of mitophagy genes will be measured in monocytes/macrophages, neutrophils and CD8 T cells in AAV patients. A fraction of the isolated cells from the previous work package will be processed for RNA extraction and cDNA synthesis. A selected panel of mitophagy genes will be assessed through qPCR. Results will lead to mechanistic insights of mitophagy mediated immune dysregulation.

In work package 4, relative weight of mitochondria and mitophagy procedure will be assessed in cell subsets of AAV patients, isolated previously in WP2. In particular, we plan to stain monocytes, neutrophils and CD8 T cells with MitoTracker™ Green measuring the cumulative cellular mitochondrial mass, through flow cytometry. The same cell subsets will be stained with JC-1 assay (MitoProbe™ JC-1 Assay Kit), measuring fluctuations of membrane potential in mitochondria through flow cytometry in order to extract information about membrane functionality and relative number of functional mitochondria. Consecutive simultaneous staining with MitoTracker™ Green and LC3 will be assessed through confocal microscopy. This experiment will visualize the engulfment of mitochondria in autophagosomes and measure their processing and destruction through autophagy. Finally, superoxide production from mitochondria will be measured through a flow cytometry-based assay.

## ANTICIPATED BENEFITS

Mitophagy plays a significant role in inflammation and autoimmunity. The results of this study will elucidate the involvement of mitophagy in AAV and add mechanistic insights in the pathophysiology of the disease. Moreover, assessment of mitophagy in different cell subsets will shed light on their role as effectors in the phenotype of the disease, linking transcriptome to function.
